# Developing an intuitive decision support system for equitable vaccine distribution during pandemics

**DOI:** 10.1038/s41598-025-01640-9

**Published:** 2025-05-10

**Authors:** Lise Boey, Hossein Baharmand, Ross Owen Phillips, Nico Vandaele, Burcu Balcik, Jan Ove Kjøndal, Asle Birkeland, Håvard Fossli, Naima Saeed, Catherine Decouttere

**Affiliations:** 1 Access-To-Medicines Research Centre, KU Leuven, Leuven, Belgium; 2 School of Business and Law, University of Agder, Grimstad, Norway; 3 Norwegian Centre for Transport Research (TøI), Oslo, Norway; 4 Department of Industrial Engineering, Özyeğin University, Istanbul, Turkey; 5 Agens, Oslo, Norway

**Keywords:** Vaccine supply chain, COVID-19, System dynamics, User-centered approach, Decision-support system, Health care economics, Information technology

## Abstract

Effective organization of the vaccine supply chain is vital to achieve high vaccination rates in pandemics. This paper presents a novel approach for developing a decision support system (DSS) to support health officials and policymakers who must make timely and impactful decisions with limited information for distributing vaccines. We combine a stakeholder-informed systems approach for problem definition with a user-centered design approach for DSS development. The methodology has been tested during the COVID-19 pandemic. We used system modeling to capture stakeholders’ knowledge, experience, and learnings from the H1N1 pandemic, leading us to focus on the central vaccine allocation problem (CVAP), which involves determining the number of vaccines allocated to each municipality in an equitable way. We designed a dashboard that embedded a mathematical model as a DSS for in-country CVAP during a pandemic. A Lightning Decision Jam workshop was conducted to define the DSS’s characteristics, with the visualization of scenarios and decision-making transparency being key features during the development process. We validated the DSS with policymakers and built it for the Norwegian context, with flexibility for adaptation to other regions, particularly those still grappling with high vaccine supply deficits. Our approach offers a novel and practical way to develop DSSs to support policymakers making critical decisions during pandemics.

**Supplementary Information:**

The online version contains supplementary material available at 10.1038/s41598-025-01640-9.

## Introduction

The coronavirus disease 2019 (COVID-19) pandemic caused more than 6.5 million deaths (situation in October 2022)^1^. Vaccines against COVID-19 have been developed at an unprecedented speed. One year after the World Health Organization announced corona-related pneumonia cases in Wuhan, 70 vaccines were in clinical testing, and the European Medicines Agency approved two vaccines^2^. For vaccines to achieve their immunization purpose, they must reach the people’s arms. Adequate organization of the vaccine supply chain is essential to attain this. Common vaccine supply chain challenges include limited shelf life, specific storage requirements, and delivery to remote areas^3^. Designing vaccine supply chains inherently involves dealing with a lot of uncertainty, for example, due to unknown upcoming epidemiological evolutions or potential disruptions in facility operations or transportation^3^. The COVID-19 pandemic increased this uncertainty even more. In the early pandemic, it was unclear what type of vaccines would make it to the market, how many doses would be needed, or what the temperature requirements would be. When vaccines became available, uncertainties remained. Vaccine delivery times were often unpredictable, and insights from clinical trials and real-world use were still evolving. The COVID-19 pandemic has challenged policymakers to deal with this uncertainty and make high-impact decisions under severe time pressure.

Important decisions made at the start of the pandemic were those regarding vaccine procurement, allocation, and distribution. Upon in-country arrival of the vaccines, public health authorities had to ensure that the limited number of available vaccines were allocated fairly and in a way that minimized disease transmission. International allocation frameworks helped countries set priority groups based on risk of infection, risk of severe morbidity and mortality, risk of infecting others, and risk of impact on the society and economy^4–6^. In many countries, however, reducing inequities between socio-economic groups, rural and urban residents, and municipalities remained challenging. In addition, organizing local distribution and transport to remote areas was difficult^7^.

Simulation and mathematical modeling are valuable tools to inform and support decision-making^8^. For example, Lee et al. used agent-based modeling to evaluate the outcomes of different vaccination scenarios during the 2009 H1N1 pandemic^9^. Sun et al. used a simulation-based approach combining route optimization and dynamic simulation to advance the coordination of the COVID-19 vaccine distribution in Norway^10^. Lemmens et al. described the importance of including stakeholder perspectives on supply chain design^3^. A systems approach is needed as a basis for models as it helps to understand the complex relationships between system components and to anticipate optimal outcomes or potential challenges in a complex context^11–13^. In COVID-19 years, related DSS work has been published based on simulation and Geographical Information Systems (GIS) for prioritising demand allocation and optimising vaccine distribution^14^. A concise literature review on quantitative methods for COVID-19 vaccine allocation and distribution focuses on equity and hesitancy^15^.

We present a novel way of understanding decision support needs and developing a DSS for organizing the supply chain in the context of the pandemic. We aimed to learn from the viewpoints of stakeholders involved in Norway’s response to COVID-19 and to propose a generic methodology that can support supply chain public health authorities in public health response in other countries.

## Methods

**Fig. 1:**
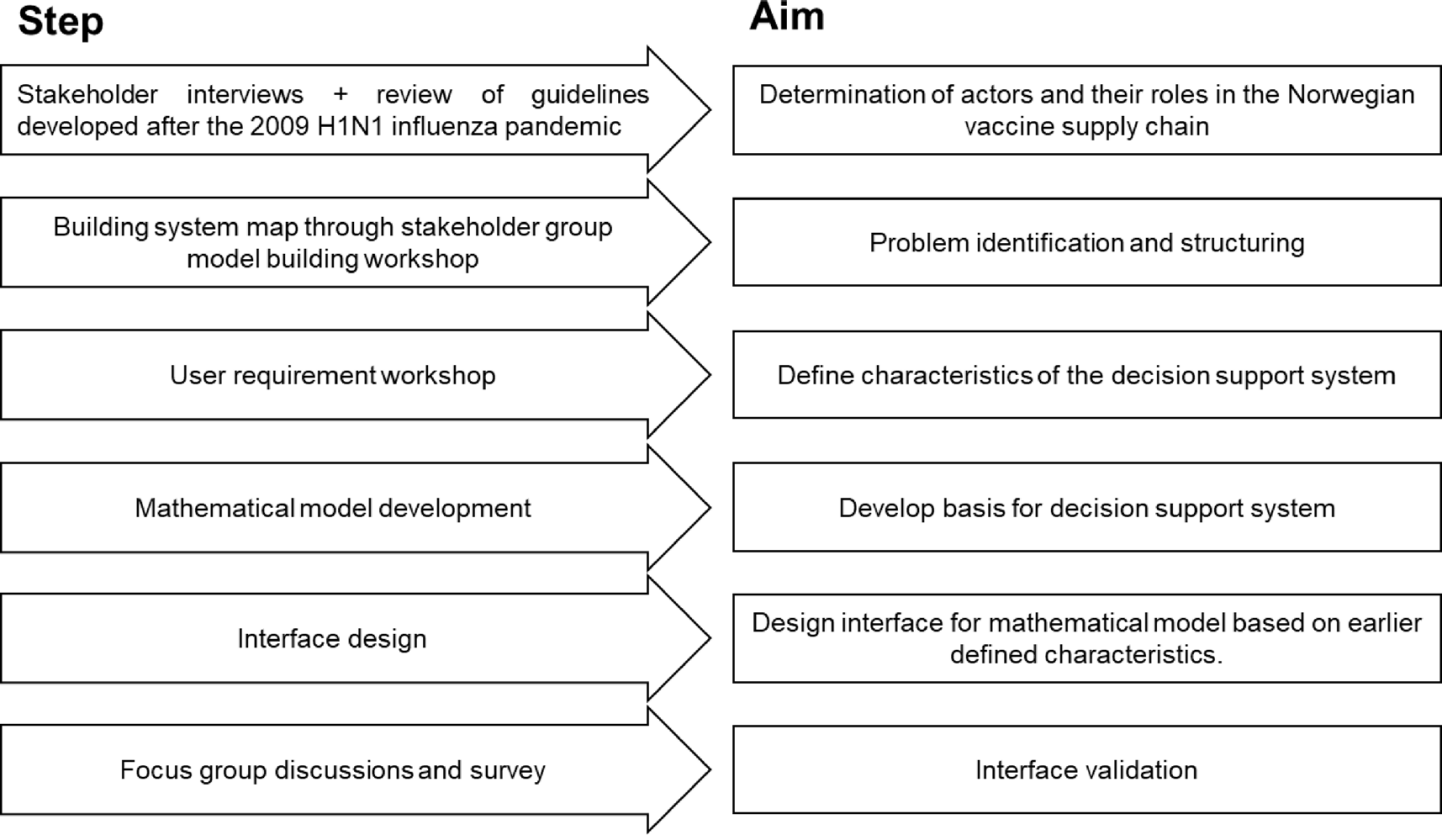
Overview of the research process. It begins with stakeholder insights, then uses those insights to define the problem and design a decision support system for it.

This study, which started in June 2020 and ended in March 2022, deploys a combination of a stakeholder-informed systems approach for problem definition and a user-centered design approach for developing the DSS^16^. We defined stakeholders as actors influencing and influenced by the organization of the vaccine supply chain and users as those who may use the DSS. Figure 1 illustrates the various steps in the process. The first step of the project is to identify the key stakeholders in the vaccine distribution network in Norway, map their interrelationships, and distinguish critical decision points in the system. To this end, interviews were performed in August and September 2020 with key stakeholders from the Ministry of Health and Care Services (HOD), Institute of Public Health (Folkehelseinstituttet (FHI)), the County Governor for Vestfold and Telemark, and the Directorate for Civil Protection (DSB). The stakeholders were selected based on their knowledge and experience of their respective organizations in vaccine distribution. With these interviews, we aimed to find relevant information and knowledge of vaccine distribution and emergency response and to identify prominent stakeholders in vaccine distribution in Norway. Information from the interviews was analyzed together with national guidelines on vaccine response procedures and evaluations of the H1N1 pandemic response in 2009^17–22^ to to develop a map of actions and information flows among stakeholders. Mapping was based on principles for mapping control structures in complex socio-technical systems^23^. The map was validated by the participants in the workshop, as described in the next paragraph.

Secondly, we defined the problem structure. To this end, we organized a group model building (GMB) workshop in October 2020 to gain further insights into the vaccine outbreak response system in Norway. GMB is a system dynamics model-building method to enable shared understanding of complex system structures, the relationship between the elements of the system, and the system’s behavior. GMB allows stakeholders to be part of the construction of the system dynamics model^24^. Stakeholders identified in the stakeholder map were invited to a virtual GMB workshop using the online collaborative and interactive platform Miro (miro.com)^25^. The workshop took five hours and covered the following four aspects of the COVID-19 system: supply side, demand side, supply meets demand, and decision-making regarding supply and demand. The workshop was organized to capture stakeholders’ views on the expected performance of the COVID-19 vaccine supply chain system based on their experience from the H1N1 pandemic. Topics covered in the discussion were (1) stakeholders’ roles, responsibilities, foreseen challenges within and outside their role (supply side), (2) priority groups, vaccine hesitancy, high transmission areas, geographical dispersion of vaccine-eligible people (demand side), (3) constraints (e.g. cold chain), actions to take in case of imbalance between supply and demand (supply meets demand), and (4) the decision maker, their area of control, system performance metrics and requirements for the decision support system (decision making). For each of these four aspects, a brainstorming session was performed and contained a brief explanation of the content, a list of guiding questions, a dedicated time to brainstorming, and a debrief. The information provided by the stakeholders was collected immediately in Miro. We followed the principle of information saturation for each debrief, which implied that each additional stakeholder only added elements that were not yet mentioned in the Miro system. Subsequently, the research team developed a system map in the format of a stock-and-flow diagram (SFD) based on the information gathered during the workshop and the interviews. An SFD is a visual representation of a system model using stocks, flows, converters, connectors, sources, and sinks^6^. The system map takes a systems view of the vaccination process from research and development to actual vaccine administration. It served as a means for problem definition for which the DSS was to be developed.

Thirdly, a user requirement workshop was organized in March 2021. It used the format of a “Lighting Decision Jam” to understand and prioritize problems to solve, foster creativity, avoid groupthink, and define the DSS’s characteristics^26^. Fourthly, we developed a decision support system that consists of a mathematical model written in Python to allocate the available vaccines to municipalities equitably and effectively. Fifthly, the dashboard interface was made on top of the model written in Python. The dashboard made it easy to input constraints and requirements while getting instant feedback on how the vaccines would be allocated. The dashboard was made to feel familiar with an Excel-like table where the decision maker can input values and get automatic calculations in other cells and graphs. For further modifications of the dashboard interface, we discussed characteristics of an effective allocation decision system with two Flemish public health officials working on COVID-19 vaccination and their allocation decision-system software developer in March 2021. In addition, we received feedback from the academic advisory board members of the project in December 2021.

Lastly, the dashboard was validated by a group of 15 experts from academia and public health authorities using a combination of 3 focus group discussions (FGD) and a short online survey, which was sent to the participants right after the discussion. The focus group discussion included an introduction to the research project, dashboard demo, test use case, and discussion. More information about the organization and participation in the validation study can be found in the supplementary material.

## Results

### Key stakeholders and roles in the Norwegian vaccine distribution network

**Fig. 2:**
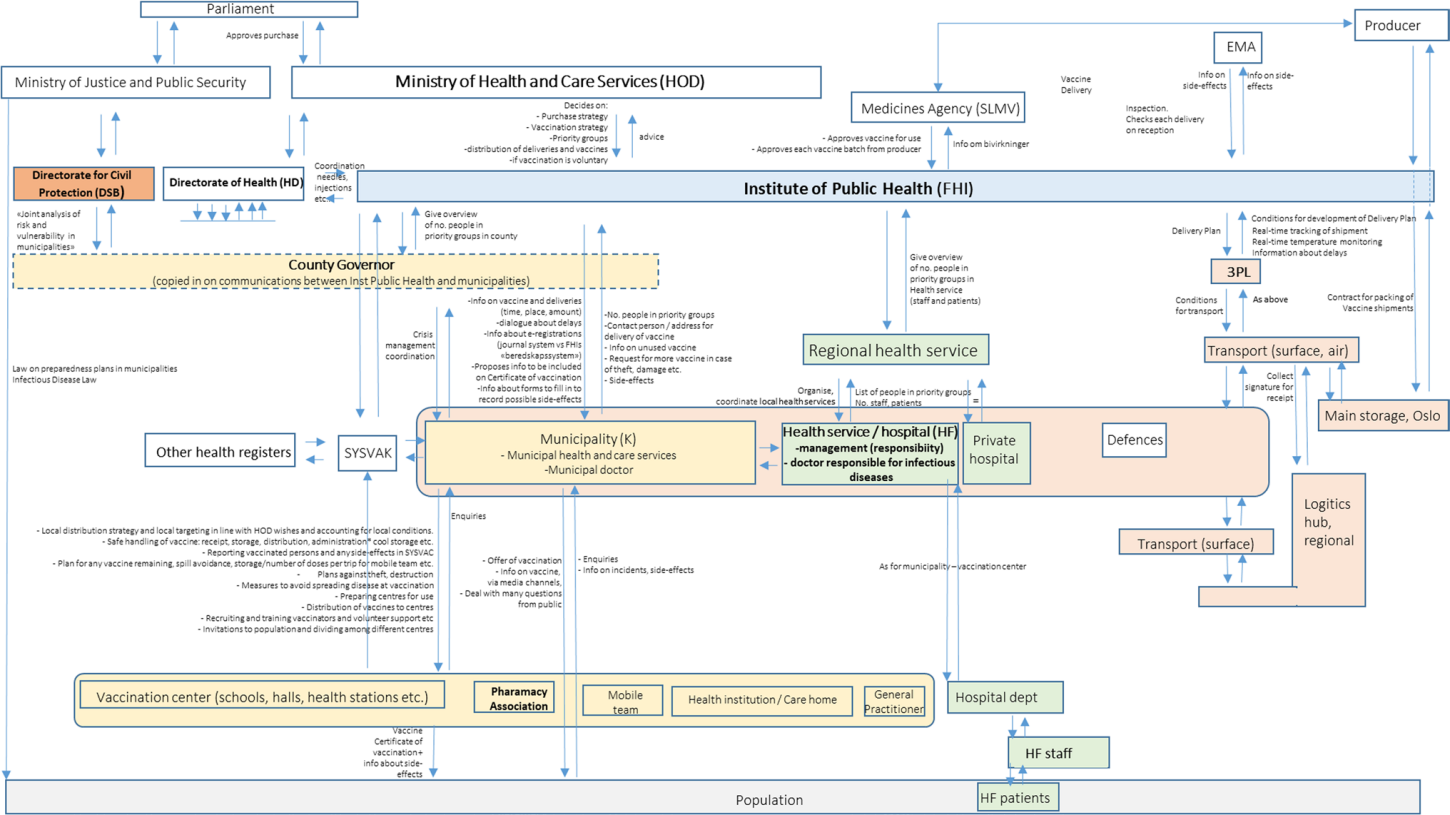
Stakeholders map. Illustration of stakeholders and stakeholder relationships involved in the distribution of vaccine in Norway. Orange boxes represent the stakeholders involved in the transport from central to regional or municipal level. Yellow boxes represent the stakeholders involved in the vaccine distribution organized by the local and regional government. Green boxes represent the stakeholders involved in the vaccine distribution organized by the local and regional health services. A downward arrow signifies purposeful action taken to influence the object (person, organization, plans, technology) described underneath the arrow. An upward arrow describes the feedback (e.g., data, awareness, knowledge) about the purposeful action that the object is taking. A sideways arrow describes information flows that do not directly targets a purposeful action. 3PL = third-party logistics company, EMA = European Medicines Agency, SYSVAK = database for registering of vaccination data. Acronyms in brackets following stakeholder names are the Norwegian acronyms in common use in Norway.

The interviews with key stakeholders revealed that, after the H1N1 pandemic 2009, new policy guidelines were developed for new (influenza) pandemics/epidemics at the national and regional levels^17–22^. These documents provide frameworks for crisis management and describe all stakeholders, their roles and responsibilities, priority groups, materials, instruments, and resources available in a pandemic. This served as background for the stakeholder map, which represents the control structure in the vaccine distribution system in Norway. This map is shown in Fig. 2 and represents the vision as it was during the workshop and does not include subsequent decisions about roles and responsibilities.

In summary, the Norwegian Parliament approves vaccine purchases and prioritization strategies formulated by the Ministry of Health and Care Services (HOD). In the case of COVID-19, a working group was set up, which included FHI and the Directorate of Health (HD). In coordination with FHI, HD arranges equipment such as syringes and needles, which are needed during the vaccination campaign. FHI is the central stakeholder in the vaccine distribution system. They also provide scientific advice to health authorities (HD, HOD) and advise on vaccine distribution plans. FHI is responsible for receiving and distributing the vaccine nationally after the vaccine is approved by the Norwegian Medicines Agency (SLMV). Using a third-party logistics company, FHI handles vaccine storage, packaging, and distribution to each of Norway’s 356 municipalities. FHI communicates directly with each municipality or local health authority and provides them with information about, among other things, vaccine delivery, vaccine characteristics, and dosage. In turn, FHI receives information on local populations (e.g., the number of people in priority groups per municipality) and vaccine efficacy and side effects data. Each municipality is responsible for recruiting and training vaccinators and organizing the administrative infrastructure. Other tasks include education about the vaccine and vaccination process, issuing vaccination certificates, and registering data in the national vaccination database, SYSVAK. Municipalities also receive and handle information and questions from their populations. Hospitals have parallel duties in organizing the vaccination of their staff and patients. The pharmacy associations are included in the map as part of the civil infrastructure. Still, they have a specific role in that they may purchase vaccines through the municipalities independently of the COVID-19 dedicated distribution. The county governor is responsible for coordinating and overseeing the distribution and administration of vaccines in the municipalities in its area and communicates with FHI on issues and strategies. Regional health authorities perform parallel duties for their local health services.

### System map: discovering critical decision points in the Norwegian vaccine distribution network

**Fig. 3:**
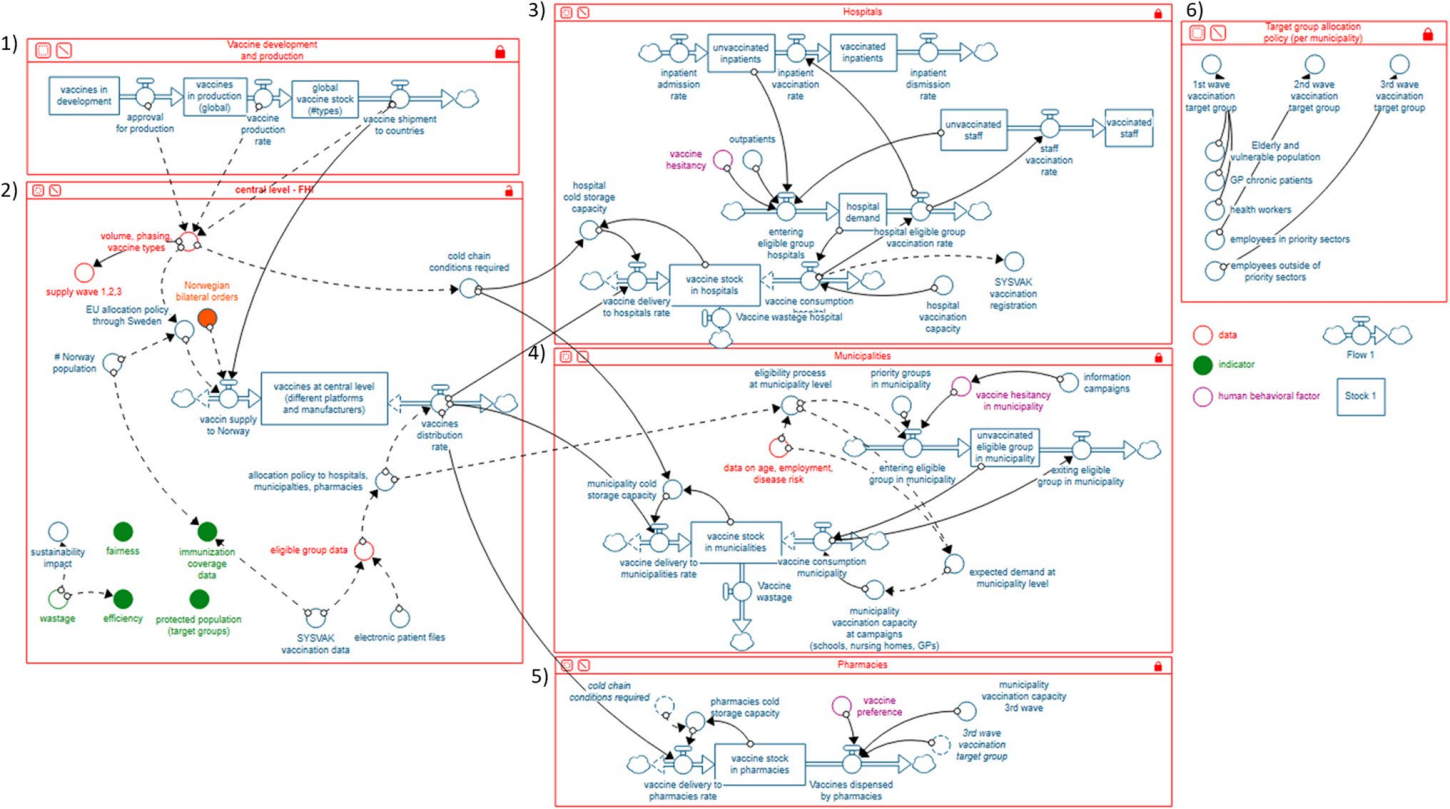
System map in the format of a stock-and-flow diagram. The map has six subsystems: (1) vaccine development and production, (2) handling of vaccines at the central level, (3) hospitals, (4) municipalities, (5) pharmacies, and (6) target group allocation. The flow icons show important inputs and outputs of stocks of data, products, or people. The circles connected to the flows by arrows are factors that affect the rate of inflow and outflow into and from stocks [24. Created with Stella Architect.

The system map (Fig. 3) was developed in the format of a stock-and-flow diagram based on the stakeholder group model building workshop and the stakeholder map. The system map represents the potential vaccine supply chain in the Norwegian context as perceived at the beginning of the pandemic. The system map was developed based on information available from stakeholders at the time. It does not reflect subsequent supply chain decisions in Norway, such as whether to involve pharmacies in the vaccination campaign. The system map has six subsystems: (1) vaccine development and production, (2) handling of vaccines at the central level, (3) hospitals, (4) municipalities, (5) pharmacies, and (6) target group allocation. The first subsystem shows the process of research and development, to manufacturing, market authorization by the Norwegian Medicines Agency, to the gradually increasing vaccine supply to FHI. The central level subsystem shows potential delivery routes to Norway and the subsequent allocation to hospitals, municipalities, or pharmacies. As shown in the map, FHI can, for this exercise, consider data concerning current vaccination uptake and the number of people prioritized. The subsystems in the hospitals, municipalities, and pharmacies show how stocks are filled with deliveries from the central level and consumed through vaccination uptake. For the organisation of the vaccine delivery, one needs to consider the expected demand, which can be forecasted for data on age, employment, and disease risk. The last subsystem shows the different waves of vaccination target groups based on FHI definitions^5^.

#### Critical decision point: central vaccine allocation problem

The vaccine supply chain system poses numerous challenges, such as identifying suitable storage and vaccination sites, allocating transportation and vaccines, and distributing vaccines to municipalities. Based on our findings from the stakeholder workshop and system mapping, we decided to focus on the central vaccine allocation problem (CVAP) in the Norwegian context. The CVAP determines the number of vaccines to be allocated to different priority groups in each municipality across the country. For the decision to focus on CVAP, we consider the decision level, the impact of the decision, and the stakeholder values and insights.

The CVAP is central to the vaccine distribution network for three main reasons, as shared by representatives from public health authorities. First, vaccines must be allocated based on different criteria. It implies considering the number of inhabitants, number of available vaccines, the current immunization level, priority groups in each municipality and storage, service delivery capacity at municipality level, and transportation. The latter is complex, depending on varying temperature requirements, the number of allocated vaccines, and the destination. In Norway, which has a low population density with an area of 385.207 km² and a population of 5.4 million, most people live in a few densely populated cities in the south and west of the country. However, the few people who live scattered across the country in smaller municipalities should not be forgotten when allocating vaccines. Vaccine distribution to those regions can be challenging at times since some areas are only reachable by ferry or via a mountain pass during the winter due to cold temperatures^27^.

Second, the impact of the CVAP on the downstream system (i.e., system delivery) is huge. As shown in Fig. 3, CVAP will determine the vaccine stock in each municipality, the number of vaccinated people, and the vaccine demand dynamics. More specifically, it also has implications for local authorities who have high power in Norway and are responsible for organizing the vaccination campaigns in Norway. It directs how they can address the challenges posed by the campaign organization. These challenges include coordinating staff and vaccine recipients with vaccine availability, integrating (changing) national policies (e.g., with respect to priority groups and dose intervals), reducing wastage, and setting up data systems^28,29^. Phillips et al. elaborate on the challenges experienced by the local authorities^29^.

Third, formulating a generally accepted strategy to address CVAP is often a complicated decision. However, everyone agrees that we must strive for equity in this decision, as no single community or vulnerable group can be ignored. Equity is a value highly important in Norwegian culture, as confirmed during our initial stakeholder workshop.

By addressing the CVAP through system thinking, we contribute to the effectiveness and equity of the COVID-19 response while accounting for the interactions between different entities in the entire system.

### Decision support tool

We developed a DSS to address the CVAP by embedding a sophisticated mathematical model^30^ in an intuitive web-based dashboard. The mathematical model and its components, such as assumptions, parameters, and decision variables, are described in detail in Balcik et al.^30^ with several numerical experiments and a case study of COVID-19 vaccine distribution in Turkey. The DSS should guide the user (e.g., decision-maker of public health authorities) to allocate the available vaccines to municipalities equitably and effectively. Underlying the dashboard development were the insights from the Lightning Decision Jam brainstorm on user requirements. Due to the high workload during the pandemic, decision-makers could not attend the workshop. Members of our interdisciplinary project team participated in the workshop. They agreed that the dashboard should clearly show the input variables and that the impact of decision scenarios should be clearly visualized.

**Fig. 4:**
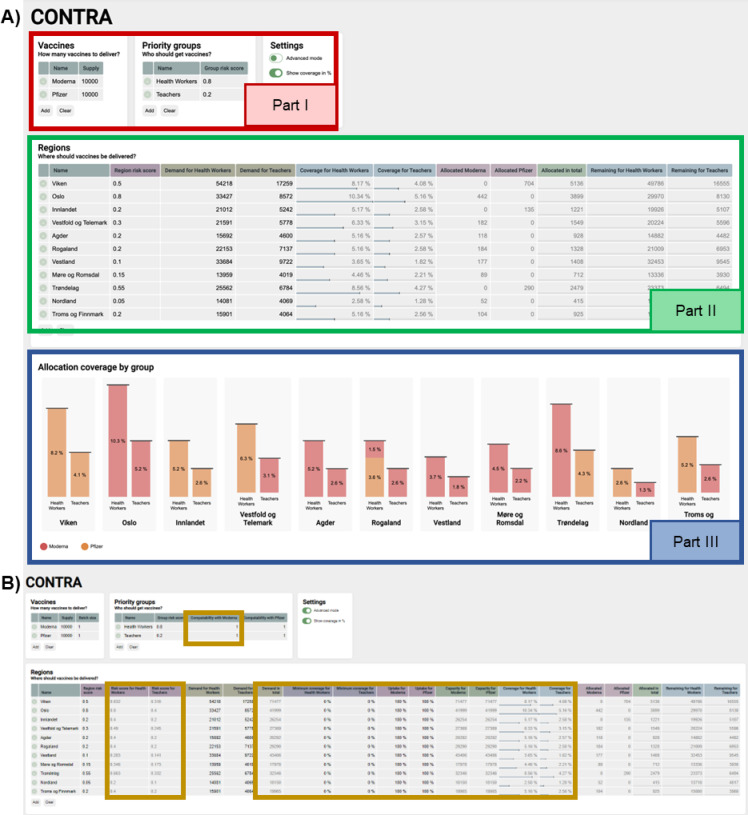
(**A**) Screenshot of online dashboard. Available at https://contra.agens.no/. (**B**) Additional fields that appear by enabling the advanced mode.

Since stakeholders had mentioned during the initial stakeholder meeting that public trust is high in Norway, we concluded that good visualization is imperative to make transparent decisions to sustain public confidence. Figure 1 in the supplementary materials shows how a user can be supported in decision-making using the dashboard. In the dashboard, one can see input variables, decision variables, and outcomes. The input variables are given, and fixed data is provided, such as the national supply of the vaccines, the municipalities, the demand per municipality, and the priority group. The decision variables are variables on which the decision-maker has an influence. In this case, this is the risk score that can be given to each municipality and priority group. The decision makers can adjust risk scores based on multiple criteria, such as socially disadvantaged regions or infection rates. Underlying the risk score determination are strategies such as achieving equity or effectiveness. The outcome section shows the results of the optimization model in terms of vaccines allocated to different municipalities and priority groups. Figure 4 shows a screenshot of the online dashboard. Part I holds the information about the vaccines and priority groups, Part II contains the information about the municipalities, and Part III includes the outcomes in graph format. The decision maker can easily add the black font input and decision variable data in parts I and II. Output data is shown in dark gray font and the graphs.

To evaluate the DSS performance objectively and provide feedback for continuous improvement, we think that multiple quantifiable metrics could be incorporated into the dashboard such as allocation efficiency, wastage minimization, and system responsiveness. Allocation efficiency could be measured through indicators such as proportion of priority groups covered, geographical equity of distribution, and alignment with epidemiological priorities^31^. Vaccine wastage, a persistent challenge in vaccination campaigns^32^, could be tracked through metrics such as doses discarded due to expiration, temperature excursions, or unused vials in opened multi-dose containers. The dashboard could also incorporate system responsiveness metrics, including time from central allocation decision to municipality delivery and the system’s ability to adapt to changing demand patterns^33^. These performance metrics would enable decision-makers to compare alternative allocation scenarios and evaluate trade-offs between competing objectives, such as speed versus equity of distribution^34^. With real-time updates as decision parameters are modified, immediate assessment of potential allocation decisions would be possible before implementation. This approach would align with WHO recommendations for monitoring vaccination campaign effectiveness^35^ and facilitate evidence-based adjustments to allocation strategies as pandemic conditions evolve.

A conversation with public health officials responsible for vaccine allocations in Flanders and insights from the literature^36^ confirmed the importance of visualizing the results of the mathematical optimization model. This way, the deviations from the intentions of the decisions can be visually anticipated. Based on these deviations, the decision-maker can return to the decision variables and adapt those to optimize the outcomes. Feedback from the scientific advisory board of the Norwegian research council and the validation study was overall positive, while some shortcomings were noted and subsequently addressed (see appendix).

## Discussion

### Implications for pandemic response and vaccine allocation

During the COVID-19 pandemic, there has been an unprecedented need for decision-makers to respond quickly with limited resources. In the case of Norway, there were already guidelines available for policy in case of pandemics or epidemics at the national and regional levels, which were developed after the 2009 H1N1 influenza pandemic^17–22^. International frameworks on priority group setting also became quickly available^4,6^. We present a novel way of understanding decision support needs and developing a DSS for organizing the supply chain in the context of the pandemic. Using a systems approach based on stakeholders’ insights, we decided to focus on the central vaccine allocation problem, which deals with in-country allocation of vaccines to municipalities, to support decision-makers with the COVID-19 response. We developed an online dashboard that integrates a mathematical model as the DSS. The DSS was validated by a group of public health officials and academics. We believe the tool helps respond to a situation where an entire population needs to be vaccinated urgently.

While developing this DSS, we gave high importance to some ethical principles that are highly valued in Norway and applied in international priority frameworks^4,6^, i.e., equity and transparency. Equity is embedded in the mathematical model, as decision-makers can adjust risk scores for municipalities and priority groups based on multiple criteria (e.g., socially disadvantaged regions, infection rate) (see Balcik et al.^30^). The DSS will also enhance the transparency of decision-making. During the user requirement workshop, the importance of clearly showing which data is used and visualizing the results was discussed. Good visualization will clarify why policymakers have taken a specific vaccine allocation strategy. Ivanković et al. also mention that visual cues are important to make a dashboard actionable^36^.

Transparency and equity are also vital in times of vaccine hesitancy^37^. Although public health stakeholders mentioned during our workshop that public trust is high and vaccine hesitancy relatively low in Norway, which is also described in the literature^38,39^, it is important to maintain this. In January- February 2021, about 10% of participants in a Norwegian online survey of about 4,500 adults showed hesitancy about COVID-19 vaccines^39^. It is important to closely monitor and address any increases in vaccine hesitancy as it is a very dynamic phenomenon that can be easily impacted by certain events or communications^40^. An example of such an event is Norway’s decision to withdraw the Oxford-AstraZeneca and Janssens vaccines for the program in March and April 2021, respectively^38^.

By May 2022, demand for vaccines in Norway no longer exceeded supply^40^, the DSS may still be applicable. Equitable allocation of vaccines is always important^7^, be it for allocating booster doses of COVID-19, newer vaccines (e.g., bivalent COVID-19 vaccines), or vaccines against other diseases. In addition, it will also be good to have the DSS on hand in case a new pandemic occurs. Having this tool alongside the existing policy guidelines and frameworks will enable a faster initial pandemic response.

We would also like to note that although the DSS was developed in Norway, we believe it is still of value for transfer to other regions and countries, particularly to those with a high vaccine supply deficit. In January 2022, 93% of adult Norwegians had already received at least one dose of a COVID-19 vaccine, 90.2% of adults received two doses, and booster shots were being administered^41^. Many other countries have not yet reached such high vaccination levels. By October 2022, for example, only 23% of the population on the African continent had completed the primary vaccination series^42^, which is mainly due to unequal access to vaccines as a result of high-income countries prioritizing their citizens over global health benefits, inadequate infrastructure for production and distribution, and misinformation about vaccines^43^. Many countries with low current vaccination rates also have to deal with a dispersed population and high power of local authorities, as was the case in the Norwegian context.

We also believe that the DSS may have applications beyond the COVID-19 pandemic. Since the African Union is putting much energy into increasing vaccine manufacturing capacity in the African continent^44^, it could be applied when the supply of these vaccines increases but has not yet reached the total vaccine demand. In this case, the DSS could assist decision-makers in allocating vaccines between regions in the continent and within the countries. Our DSS was designed for easy adaptability to the other areas, allowing data input to be easily replaced with data from different countries. Further research will have to be done to confirm this convenience.

## Customizing the DSS across different contexts

The DSS developed in this study was designed with adaptability in mind, allowing for customization based on local contexts and constraints. We propose a three-dimensional framework to guide the adaptation process for other countries or regions.

**Fig. 5:**
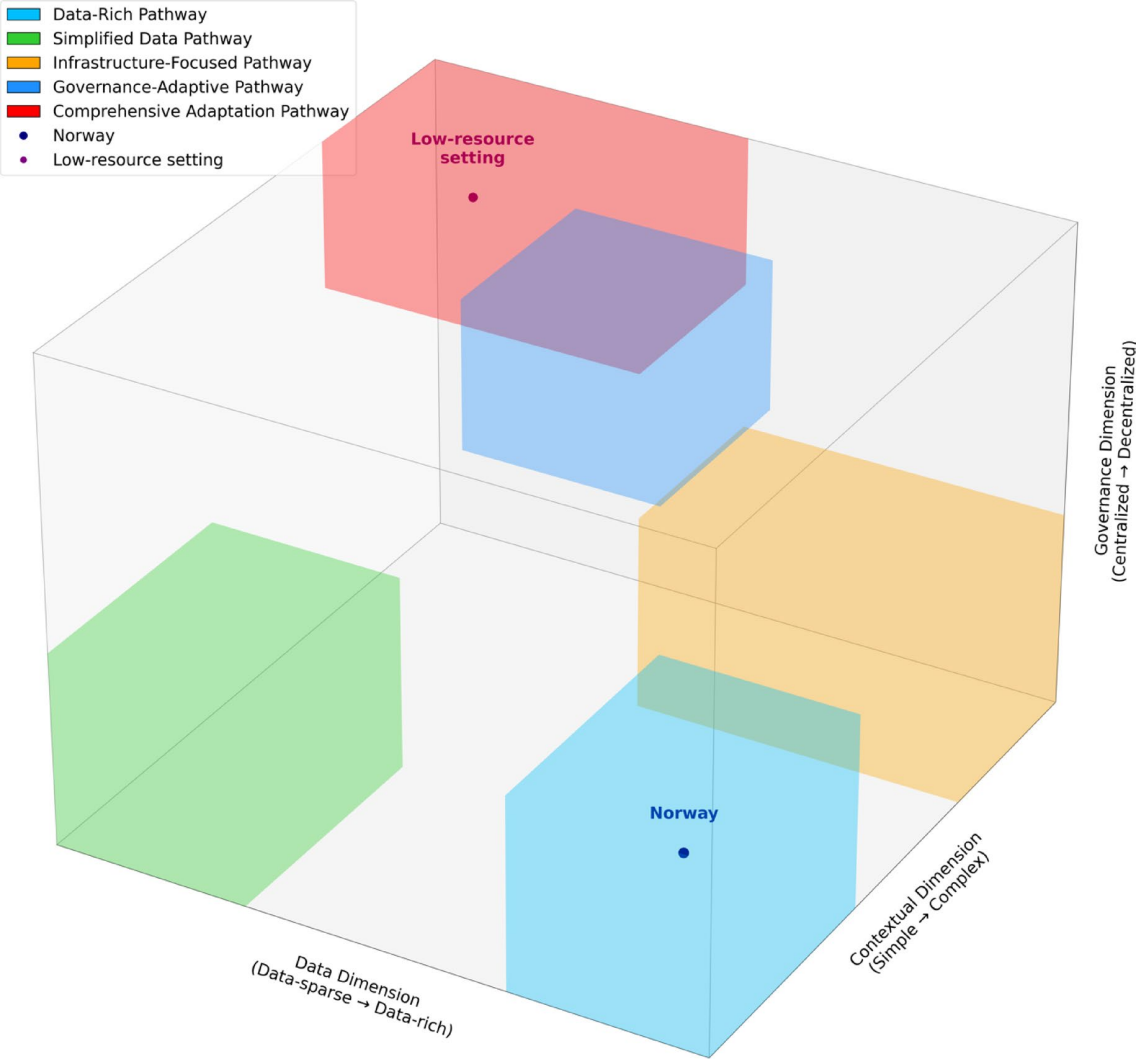
Illustration of the proposed framework for DSS customization across different contexts.

Figure 5 illustrates the three-dimensional framework for adapting the DSS to different settings. The data dimension (x-axis) ranges from data-sparse to data-rich environments. The model requires certain data inputs, but data availability varies considerably across contexts. In high-resource settings with robust health information systems, detailed demographic and epidemiological data can be incorporated. In settings with limited data infrastructure, the model can be simplified to operate with aggregated data or proxies. For example, when detailed risk factor data is unavailable, broader demographic categories can be used^45^. Ozawa et al.^46^ demonstrate that even in data-sparse environments, simplified epidemiological models can guide effective vaccine allocation.

The contextual dimension (y-axis) represents the spectrum from simple to complex geographical, demographic, and infrastructure challenges. Local demographics, geography, and existing healthcare infrastructure significantly impact vaccination campaigns. The dashboard allows adjustment of risk scores to account for these contextual factors. Urban-rural disparities, transportation networks, and cold chain capabilities can be incorporated as constraints in the model^47^. In mountainous regions or areas with seasonal accessibility challenges (similar to Norway’s remote areas), transportation constraints can be weighted more heavily^48^.

The governance dimension (z-axis) spans from centralized to decentralized decision-making structures. Decision-making structures vary across countries, from centralized to highly decentralized systems. The DSS can be configured to reflect these governance structures by adjusting the geographical units of allocation (e.g., regions, districts, municipalities) and incorporating different levels of decision-making authority. Lloyd et al.^49^ highlight that vaccine allocation tools must align with existing governance structures to be successfully implemented.

The colored areas represent different adaptation pathways depending on where a specific setting falls within this three-dimensional space. Implementation complexity increases as contexts move toward higher complexity across multiple dimensions simultaneously. This framework aligns with “contextual intelligence” in global health implementation^50^ — the ability to adapt evidence-based tools to local realities while maintaining their core functions.

The three dimensions (data availability, contextual factors, and governance structures) create a space where different countries can be positioned based on their characteristics. For each position in this space, we recommend specific customization strategies:

- **For data-rich environments** (like Norway): Focus on leveraging detailed health information systems to enable highly granular allocation decisions. Incorporate detailed risk factors, real-time vaccination rates, and integration with existing health databases to support municipality-level allocation decisions based on multiple priority criteria.
- **For contexts with limited data**: Simplify the model’s data requirements by using proxy indicators and broader demographic categories. For instance, use age as the primary risk factor rather than detailed comorbidity data, or aggregate geographical units for allocation decisions. This approach prioritizes robust implementation over granular precision.
- **For areas with geographical challenges**: Emphasize transportation constraints and cold chain limitations in the allocation algorithm. This might involve adjusting delivery schedules based on seasonal accessibility or prioritizing allocation to areas with appropriate storage capabilities while developing alternative distribution strategies for remote regions.
- **For decentralized governance systems**: Adapt the DSS to accommodate different decision-making authorities at various administrative levels. This might involve creating nested allocation models or developing interfaces that support collaborative decision-making across governance levels.
- **For contexts with challenges across all dimensions**: Implement a phased approach, starting with core functionality and progressively adding complexity as capacity develops. Begin with a simplified version of the DSS focused on the most critical allocation decisions, with planned evolution as data systems, infrastructure, and governance structures mature.

Norway’s position in this framework would be characterized by strong data systems, significant geographical challenges due to its dispersed population, and well-established local governance with significant municipal authority. This positioning requires customization that prioritizes detailed data analysis while accounting for transportation challenges to remote areas.

That said, we propose that to customize the DSS for other contexts, a structured approach could be followed: (1) Assessment of available data sources and quality; (2) Mapping of key contextual constraints; (3) Analysis of decision-making processes and authority levels; (4) Configuration of the DSS based on the intersection of data availability, contextual factors, and governance structures; (5) Validation with local stakeholders.

### Limitations and future directions

While expert input informed development, the dashboard was not tested in real-time, leaving its practical robustness unconfirmed. The model does not yet incorporate dynamic feedback, past allocations, or behavioral factors, which may affect real-world accuracy. Due to pandemic-related constraints, decision-makers could not directly join the Lightning Decision Jam, which limited opportunities for user input. Future work should incorporate a system dynamics component to enable feedback loops between implemented decisions (e.g., vaccination uptake by municipality and priority group) and input data, improving the DSS’s accuracy in dynamic contexts. An ethical impact assessment is also recommended to identify potential biases and ensure alignment with global health equity principles. Finally, comparative studies across diverse geographic and healthcare settings are needed to assess the DSS’s adaptability and generalizability.

## Conclusion

The DSS, a dashboard with an integrated mathematical model, was developed to support decision-makers in enhancing the resilience and responsiveness of national vaccine supply chains during the COVID-19 pandemic and potentially in future pandemics. Such a DSS, built using lessons from the COVID-19 pandemic response in Norway, might be useful in preparing for the next pandemic and transfer to other regions and countries, particularly those with a high vaccine supply deficit.

## Electronic supplementary material

Below is the link to the electronic supplementary material.

Supplementary Material 1

## Data Availability

The data that support the findings of this study are available from the corresponding author, Dr. Hossein Baharmand, upon reasonable request.
