# Supplementary material for: Developing an intuitive decision support system for equitable vaccine distribution during pandemics
Source: Sci Rep. 2025 May 10;15:16339. doi: 10.1038/s41598-025-01640-9 (PMC12065852; doi:10.1038/s41598-025-01640-9)
Supplement: Supplementary file 1 — Supplementary Material 1 [file 41598_2025_1640_MOESM1_ESM.pdf]

Supplementary Information

Developing an intuitive decision support system for equitable vaccine distribution during pandemics

Lise Boey[1], Hossein Baharmand*[2], Ross Owen Phillips[3], Nico Vandaele[1], Burcu Balcik[4], Jan Ove Kjøndal[5], Asle Birkeland[5], Håvard Fossli[5], Naima Saeed[2], Catherine Decouttere[1]

1 Access-To-Medicines Research Centre, KU Leuven, Leuven, Belgium

2 School of Business and Law, University of Agder, Grimstad, Norway

3 Norwegian Centre for Transport Research (TøI), Oslo, Norway

4 Department of Industrial Engineering, Ozyegin University, Istanbul, Turkey

5 Agens, Oslo, Norway

*corresponding author; Jon Lilletuns vei 3, 4879 Grimstad, Norway hossein.baharmand@uia.no

Validation study methodology

Over three months, we arranged three validations of FGD. The first one was held on February 28th, 2022, in hybrid format with three experts: an expert from FHI and a previous director of the Norwegian Ministry of Health (both physically) and one GAVI consultant (digitally). The second and third workshops were held on March 14th and 15th, 2022 digitally. The following experts (12 in total) joined the workshops through Skype: a senior advisor and a senior manager at the U.S. CDC , a senior advisor, a policy advisor and a senior technical officer at the Africa CDC, a public health advisor from Uganda, a research analyst and a senior manager from the WHO regional Office for Africa, an executive director at public health agency of Canada, a supply chain advisor at Johnson & Johnson, a consultant and a logistics specialist at UNICEF Ethiopia. Due to the residence of participants in different time zones (U.S and Canada vs Africa and Europe), it was almost impossible to gather everyone in one workshop therefore we opted for two digital workshops in two days. The agendas for the digital workshops were similar and included: introduction to the research project, dashboard demo, test use case, and discussion. For the use cases, we used a dataset derived from vaccine distribution in Norway, as described in Appendix figure 2.

Moreover, we used the standard service from Survey Monkey for the quantitative validation study. The survey included 10 questions (multiple choice and free text boxes) about the experience using the dashboard (see appendix figure 3). The survey was developed after two validation rounds. The idea was to ensure that all questions are easily understandable and could be answered by the knowledge that a participant could get after visiting the dashboard website. Two associate professors at the University of Agder and two practitioners helped us to validate the survey. Modifications were implemented on the survey through language editing, sharpening of the questions, changing the type of

questions, revising the scales, etc. The consistency of questions was also checked, and the final version was built through the SurveyMonkey paid subscription service. The validation study was approved by the Norwegian Centre for Research Data AS (NSD) and the participants gave informed consent.

Validation study results: Shortcomings and adaptations

Fifteen people participated in one of the three FDGs and 12 of them completed the survey within the due time of 3 days. The following shortcomings of the DSS interface were mentioned:

- Vaccine uptake for each municipality could be added.
- Toggling between absolute numbers and percentage could help specifically for demand input.
- One expert noted that vaccine compatibility could be added to the system. This would be useful in the case where a specific vaccine has for example age restrictions.
- The shelf life of vaccines could be considered to be added.
- The supply of vaccines could be connected to real-time information sources from the central warehouse
- The dosing regimen could be added to the system. One participant informed that if there was scarcity of vaccines in a given country, two scenarios could be considered; i) 1 dose regimen meaning that vaccines will be allocated as they are, and ii) 2 (or more) doses regimens refer to dividing the supply into two (or more) and allocate based on the number of individuals.

Based on the comments, we added several features to the dashboard and revised the placement of various parts, as depicted in Figure 4A. For example, Part I was moved to the upper part of the dashboard. This simplified inputting the data and followed the logic of inserting input data first and receiving results afterwards. In the revised version of the DSS, Part I included input fields for names and types of vaccines that will be distributed, and priority groups with associated risk score (i.e., their relative importance weight between 0-1).

In the Settings of Part I, two options were provided to users. First, users may choose to view numbers (e.g., demands) in percentages instead of absolute numbers. Users may also select to view the dashboard in Advanced mode, which enables detailed input data (cf. Figure 4B These details were specifically derived from the comments. By enabling the Advanced mode, users would be able to decide whether there is any vaccine compatibility concern with respect to priority groups (for instance if elderly people could not receive vaccine A, the user would assign a value of 0 to this group in front of the given vaccine). Part II (cf. Figure 4A) holds input information about the municipalities. In the revised version, users could account for further details in municipalities. For instance, the risk score for each group in every municipality could now be modified. Moreover, as requested by experts, vaccine uptake for municipalities have been added and could be adjusted given the information from municipalities.

Supplementary figures

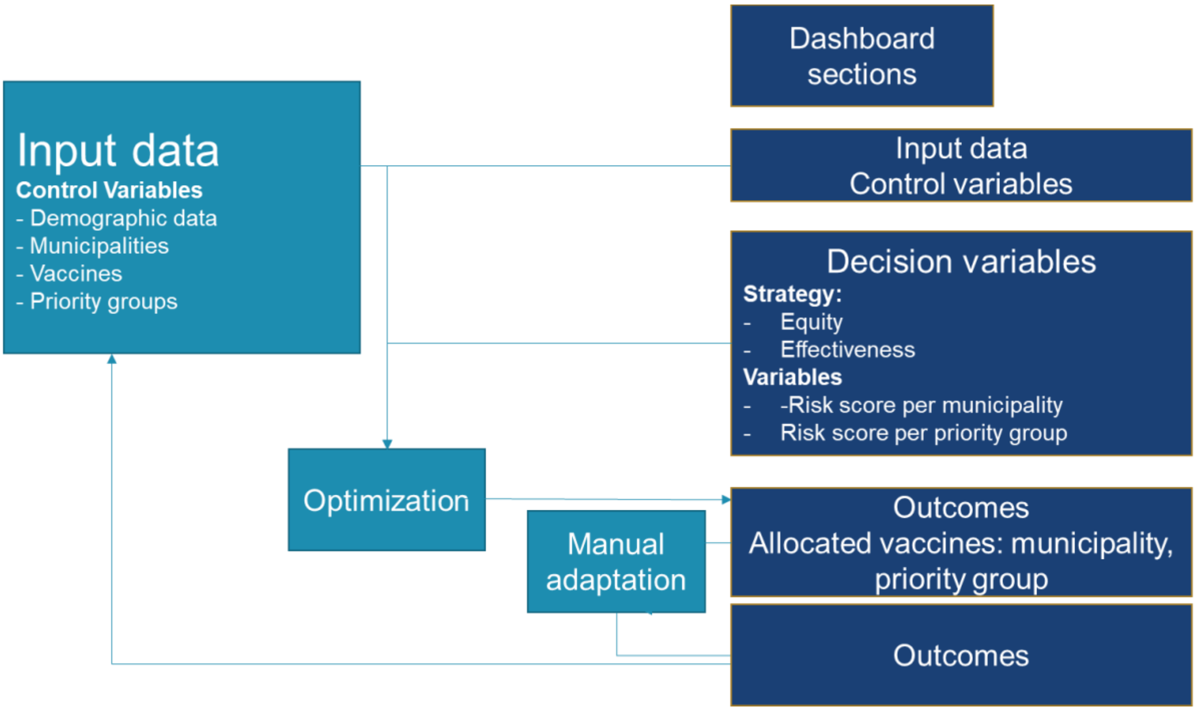

Appendix figure 1: Dashboard user flow: the way a user can be supported in decision-making using the dashboard

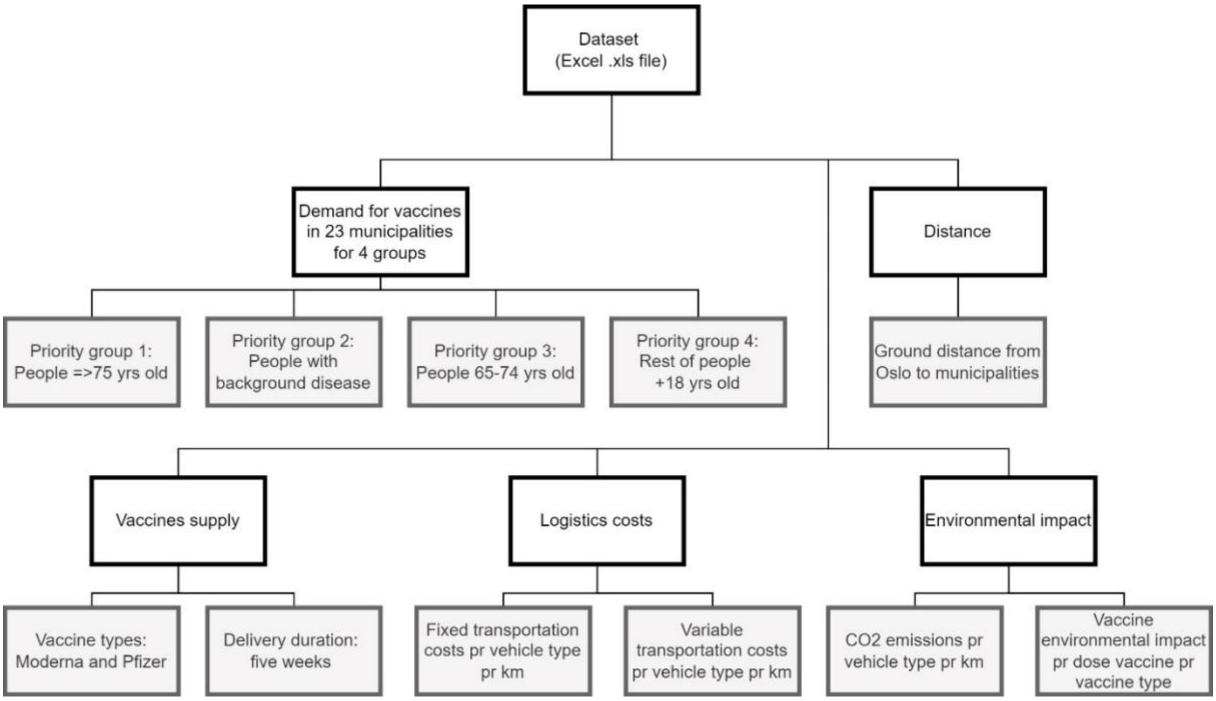

Appendix figure 2: Input data and related parameters of case study used during validation

CONTRA DSS Feedback Survey

Dear participant,

Thank you for participating in the CONTRA testing and validation workshop. We would like to ask you 10 brief questions about your impressions of the DSS, its function and dashboard for our research purposes. We will share the survey results with you in a few days.

Kind regards,
Hossein Baharmand
CONTRA project manager

1. From 1 to 5, how useful the tool was in supporting you for allocating vaccines equitably?

- ○ Definitely useful (5)
- ○ Very useful (4)
- ○ Fairly useful (3)
- ○ Slightly useful (2)
- ○ Not useful (1)

2. How satisfied are you with the results of the model (behind the dashboard) that calculates allocation quantities?

0 Satisfaction score? 100

3. From 1 to 5, how visually appealing is the dashboard?

- ○ Extremely appealing (5)
- ○ Very appealing (4)
- ○ Somewhat appealing (3)
- ○ Not so appealing (2)
- ○ Not at all appealing (1)

Other remarks (please specify) ______

4. From 1 to 5, how easy (5) or difficult (1) was it to find what you were looking for on the dashboard?

- ○ Very easy (5)
- ○ Easy (4)
- ○ Neither easy nor difficult (3)
- ○ Difficult (2)
- ○ Very difficult (1)

Other remarks (please specify)

5. Which, if any, of the issues below have you encountered during your experience with the dashboard? (Select all that apply)

- ☐ The dashboard malfunctioned
- ☐ The dashboard was missing features I needed
- ☐ The dashboard was confusing to use
- ☐ The dashboard was visually unappealing
- ☐ The dashboard crashed
- ☐ I did not experience any problems
- ☐ Other (please specify)

6. Please describe the issues you encountered in more detail.

7. From 1 to 5, how informative is the DSS in terms of visualization (figures, charts, tables, etc.)?

- ○ Definitely informative (5)
- ○ Very informative (4)
- ○ Fairly informative (3)
- ○ Slightly informative (2)
- ○ Poor (1)

What figure or chart would you change or add, if you had the opportunity to? Please specify

8. How likely is it that you would use CONTRA DSS in your work?

0 100

9. What improvements would you make to the design of the dashboard and DSS?

10. Which country do you live in, what is your position, and how long have you been in your current position? (survey control question)

Appendix figure 3: Validation survey
